# Collectanea Medica, Consisting of Anecdotes, Facts, Extracts, Illustrations, Queries, Suggestions, &c.

**Published:** 1815-09

**Authors:** 


### COLLECTANEA MEDICA,

CONSISTING OF

### ANECDOTES, FACTS, EXTRACTS, ILLUSTRATIONS, QUERIES, SUGGESTIONS, &c.

RELATING TO THE

*History or the Art of Medicine, and the Auxiliary Sciences.*

---

### *Some Account of the late* SMITHSON TENNANT, Esq.

WE announced in a former Number the death of Smithson Tennant, Esq. F.R.S. Professor of Chemistry in the University of Cambridge; we shall now proceed to lay before our readers some account of his life, studies, and character.

Smithson Tennant was the only child of the Rev. Calvert Tennant, younger son of a respectable family in Wensley-dale, near Richmond, in Yorkshire, and Vicar of Selby in that county, where Mr. Tennant was born on the 30th of November, 1761. His mother, whose maiden name was Mary Daunt, was the daughter of a surgeon of the same town.

Of his father little is known, except that he had been a fellow of St. John's College, Cambridge, and was a friend of Dr. Rutherforth, Regius Professor of Divinity in that University. He was always mentioned by his son with the most affectionate gratitude for the care he had bestowed on his education. To this he appears to have devoted himself from his son's earliest infancy; since he began to instruct him in Greek when he was only five years of age.

He had the misfortune to lose his father when he was about nine years old; and, before he attained the age of manhood, his mother was thrown from her horse, whilst riding with her son, and killed on the spot.

Mr. Tennant's education subsequently to his father's death was irregular, and apparently somewhat neglected. He was sent successively to different schools in Yorkshire, at Scorton, Tadcaster, and Beverley. He is described by one who recollects him at the first of these places as of a grave and pensive cast, with the appearance of being indolent and dispirited, rarely joining in the amusements ments of the rest of the boys. Being an only child, and under little restraint with his mother, he appears to have left home with singular reluctance, and to have had little enjoyment while at school. There is reason to believe that he looked back upon this period with no agreeable recollections, since he very seldom alluded to the events of his early life; and it is in the recollection of the writer of this narrative that, on reading Mr. Gibbon's Memoirs, he entirely concurred in the protest which the historian has entered against the "trite and lavish praise of the happiness of our boyish years."

His talents, if they had been known, would scarcely have been understood, by those concerned with his education. He appears, indeed, to have been little indebted for the eminence which he afterwards obtained, to any of his various instructors, and may be considered in a great measure as *self-educated*. This must be more or less true of every person of distinguished talents or vigorous understanding. That it was in a remarkable degree the case of Mr. Tennant, will be evident from the few anecdotes, which are now recollected, of his early life.

He gave many proofs, while very young, of a particular turn for chemistry and natural philosophy, both by reading all books of that description which fell accidentally in his way, and making various little experiments, which the perusal of such books suggested. His first experiment (as he has himself related) was made at nine years of age, when he prepared a quantity of gunpowder for fire-works according to directions contained in the Encyclopedia, or some other scientific book to which he had access.

During the time he was at school at Tadcaster, he happened to be present at a public lecture given by Mr. Walker, formerly well known as a popular teacher of experimental philosophy. Although then very young, he put several pertinent questions to the lecturer respecting some of the experiments, and displayed so much intelligent curiosity as to attract the attention of the audience, and give great additional interest to the lecture. Mr. Walker was so sensible of the effect which the boy's presence had produced, that he requested he would continue to attend during the remainder of the course.

From Tadcaster, Mr. Tennant was removed to Beverley, then rather a considerable school, under the care of Dr. George Croft, who afterwards obtained ecclesiastical preferment at Birmingham, and became known as a controversial writer. Mr. Tennant went somewhat late to Beverley, and did not readily enter into the studies or discipline of the place. But, although he was singular in his habits, and led rather a sequestered life for a school-boy, he was very far from being idle. There was fortunately a good library belonging to the school, containing a great collection of miscellaneous books, to which he devoted as much time as was in his power. His studies, even at that early period, were principally directed to works of natural philosophy; and Sir Isaac

 Newton's

Newton's Treatise on Optics was one of the books which he read with the greatest eagerness.

About the time of quitting school he was very desirous of completing his education under Dr. Priestley, whose reputation, in consequence of his brilliant pneumatic discoveries, was then at its height. His mother seems to have been disposed to gratify him in this particular; but the design was found to be impracticable, in consequence of Dr. Priestley's other engagements.

With such tastes and habits, it cannot be supposed that Mr. Tennant, at the time of leaving school, was a very regular or accurate classical scholar. Yet for every really useful purpose he possessed the full advantages of a classical education. He had a competent knowledge of Greek, and was well versed in the Latin language. What was still more important, he had acquired a strong feeling, and rational admiration, of the great writers in those languages, whom he justly regarded as the standards of true taste, and models of literary composition; and he continued during the whole of his life to be a diligent reader of the principal Latin Classics.

In the choice of a profession, his attention was naturally directed to the study of medicine, as most nearly allied to his philosophical pursuits. About the year 1781, having fixed himself at Edinburgh, he had the good fortune to meet with an instructor in the celebrated Dr. Black, well calculated to direct his curiosity.

Of his companions, studies, or occupations at Edinburgh, nothing particular is known. His stay, indeed, at that university, was of no long continuance; for in October, 1782, he was admitted a member of Christ's College, Cambridge, where he began from that time to reside. He was at first entered as a pensioner; but, disliking the ordinary discipline and routine of an academical life, he obtained an exemption from those restraints by becoming shortly afterwards a fellow commoner.

It was at this period that he began to be intimately connected with Sir Busick Harwood, the late professor of anatomy at Cambridge, then also a fellow commoner of the same college. During a long residence in the university, Professor Harwood was familiarly known to a very extensive circle of acquaintance, by some of whom he might be chiefly valued for his social and convivial qualities. He had other merits, however, of a much higher order; and at the time when he first became acquainted with Mr. T. some circumstances connected with his history and situation gave a peculiar interest to his character.

Sir B. Harwood had gone early in life to the East Indies, where he had obtained a competent fortune as a surgeon. Being compelled by ill health to return to his native country, he lost, by the misconduct of an agent, nearly the whole of what he had acquired. With the most cheerful and manly firmness, he began again his career of life; entering at Cambridge at an advanced age for the purpose of obtaining a medical degree. His misfortunes and the spirit with with which he rose above them, added to his liberal and benevolent disposition, his practical skill in medicine, his knowledge of anatomy and physiology, and his interesting accounts of the remote countries in which he had lived, produced their effect upon an ingenuous and inquisitive mind; and although there was a great disparity in the ages of Mr. Tennant and Professor Harwood, and a considerable difference in their tastes and habits, a cordial and sincere friendship was soon formed between them.

Having entered somewhat late at Cambridge, and being destined for the medical profession, Mr. Tennant did not pay any great attention to the regular course of academical reading, or devote much of his time to the study of mathematics. He acquired, however, a general knowledge of the elementary parts of that science, and made himself master of the most important propositions in Newton's Principia. But his attention was principally directed to chemistry and botany; and it may be recorded as an instance of his early progress in the former science, that even then he mentioned to some of his friends the substance of an experiment respecting heat, which he did not make public till more than twenty years afterwards. The experiment consisted in a mode of effecting a double distillation by the same heat; which forms the subject of his last paper published in the Philosophical Transactions, and which we have given in a former Number.

The attention of the chemical world was at this time principally engaged by the great controversy respecting the antiphlogistic theory, which experienced much opposition in England. It may perhaps be worth mentioning that Mr. Tennant entirely satisfied himself as to the truth of this doctrine, when at Cambridge, at a very early period, and long before it obtained a general reception in this country.

Whilst engaged in these scientific pursuits, he was at the same time a very general reader of all the most interesting works in polite literature, history, metaphysics, and especially in political economy, which was one of his favourite studies, and on which he had already made many just and original observations. Although thus incessantly employed, there was a singular air of carelessness and indifference in his habits and mode of life; and his manners, appearance, and conversation, were the most remote from those of a professed student. His college rooms exhibited a strange disorderly appearance of books, papers, and implements of chemistry, piled up in heaps, or thrown in confusion together. He had no fixed hours or established habits of private study; but his time seemed to be at the disposal of his friends; and he was always ready either for books or philosophical experiments, or for the pleasures of literary society, as inclination or accident might determine. But the disadvantages arising from these irregular habits were much more than counterbalanced by extraordinary powers of memory and understanding, which enabled him to retain whatever was most interesting and valuable in the books he perused.

It

It was during Mr. Tennant's residence at Cambridge that his principal friendships were formed; and the recollection of those who best knew him, will dwell upon this happy period of his life with a fond and melancholy pleasure. His health was then vigorous, his spirits constant and unwearied, and his talents for society more striking and brilliant than even in his after-years. He was distinguished, at that early period, by an extent of information, and maturity of judgment, which might have seemed to be the results of a long life of study and reflection; and these extraordinary attainments derived an additional interest, and peculiar grace, from the simplicity of his manners, the playfulness of his wit, and the careless, fascinating beauties of his conversation!

The summer of 1784, was employed by Mr. Tennant in travelling into Denmark and Sweden, partly to examine the great mines for which the latter country is remarkable, but principally for the purpose of visiting the celebrated Scheele, for whom he had conceived a high admiration. He was much gratified by what he saw of this very eminent person; and was particularly struck with the simplicity of the apparatus by which his great experiments had been performed. On his return to England he had a great pleasure in shewing to his friends at Cambridge various mineralogical specimens with which Scheele had presented him, and in exhibiting to them several interesting experiments which he had learned from that philosopher.

His next journey to the continent, a year or two after his Swedish expedition, was to Paris, where he became acquainted with some of the most considerable French chemists. During his stay in that city he was seized with a dangerous illness, which gave his friend, Professor Harwood, an opportunity of shewing the warmth of his friendship. He repaired instantly to Paris, but, finding his friend nearly recovered, he accompanied him on a tour through Holland and the Netherlands, previous to their return to Cambridge.

The latter of these countries, at the time when it was visited by Mr. Tennant, was in a state of insurrection against the Emperor Joseph II. and exhibited the singular spectacle of a bigotted people resisting a philosophic tyrant, and contending for their ancient privileges and establishments with the zeal and ardour of an enlightened nation.—Holland, then free and prosperous, presented a scene still more interesting and congenial to Mr. Tennant's feelings. He saw in that extraordinary country a striking illustration of his own most favourite opinions. He was gratified by the triumph of intelligent and persevering industry over the greatest physical difficulties; and by the general diffusion of wealth and comfort, the natural effects of unrestrained commerce, and of civil and religious liberty.

Such were Mr. Tennant's voluntary pursuits and occupations whilst in the prime and vigour of life, possessed of a competent fortune, exempt from every species of controul, and left to the sole guidance of his own disposition and understanding. After his mother's mother's death, which happened about the time when he went to Edinburgh, he had no near relations, and appears to have been entirely separated from all family connections. His college vacations (except when he was travelling) were passed with an intimate friend in North Wales.

On the 13th of January, 1785, he was elected, at a remarkably early age, a fellow of the Royal Society. Among the signatures to his certificate of recommendation were those of the most distinguished members of that body, who were connected with the university of Cambridge; namely, Dr. Waring, Dr. Milner, Dr. John Jebb, Dr. Maskelyne, and the Bishop of Llandaff. Such names could not but have their influence, as with most of them Mr. T. was well acquainted. With Dr. Milner he lived on terms of some intimacy.

He had hitherto continued to reside at Christ's College from the time of his entering there in the year 1782; but, Professor Harwood having for some reason determined to quit that society, Mr. Tennant removed with him in December, 1786, to Emmanuel College, of which he continued ever afterwards a member. In the year 1788, he took his first medical degree as bachelor of physic, and soon afterwards quitted Cambridge for London.

In the year 1791, he communicated to the Royal Society his analysis of the carbonic acid. M. Lavoisier had proved by decisive synthetic experiments that fixed air was a compound of oxygen and charcoal; but no one had yet resolved that gas into its simple elements.

The ingenuity and elegance of Mr. Tennant's experiment established his reputation as a chemist; and there being at the close of that year the prospect of a vacancy in the Jacksonian Professorship at Cambridge by the resignation of Dr. Milner, he was prevailed upon by his friends to become a candidate; but desisted from the pursuit on finding that he had no reasonable prospect of success.

In the year 1792, he again visited the continent, with the intention of travelling through France to Italy, and arrived at Paris not long before the memorable 10th of August. He hardly recognized some of his old scientific friends, now become members of the legislative assembly, and deeply implicated in the revolutionary politics of the times. From various circumstances, he anticipated some great and speedy convulsion, and was fortunate enough to quit Paris on the 9th of August, before the flame actually broke out.

In passing through Switzerland, he visited Mr. Gibbon, at Lausanne, and was much struck with his powers of conversation, with the sagacity of his remarks on the course and progress of the French revolution, and on the probable issue of the invasion of France by the allied armies under the Duke of Brunswick.

In Italy, he was delighted with the softness and beauty of the climate, the luxuriance of the vegetation, and astonished by the wonders of ancient and modern art at Rome and Florence. He had had hitherto been somewhat sceptical as to the degree of merit really belonging to the great masters in painting, whose fame he had supposed to be founded principally upon exaggeration. But he was converted from this error by the great works of Raphael and Correggio; and of the former he was ever afterwards a devoted and enthusiastic admirer. Returning from Italy through a part of Germany, he was much amused with the mixture of science and credulity which he found in some of the German chemists. Even the philosopher's stone was spoken of with respect.

Soon after Mr. T.'s return from the continent, he took chambers in the Temple, which continued from that time to be his established residence; and for many years his society was limited to a small circle of friends. Owing to accidental circumstances, his early connections had been much more formed among students of the law than among those of the profession which he had originally designed to pursue, but to which he was gradually becoming more and more indifferent. He had not, however, as yet abandoned the intention of practising medicine; and for several years applied himself to the cultivation of the studies connected with that science, and attended regularly at some of the principal London hospitals. Of his industry and perseverance in this course sufficient proofs exist in the medical notes and memoranda now found among his papers; and it is well known to some of his friends that he had also read with great attention most of the standard books in that science. Among these he always spoke of the works of Sydenham (with reference to the age in which they were produced) in terms of the highest admiration. Curiosity had also led him to examine the principal medical writers of antiquity, whose merits and defects he correctly appreciated, and upon whom he had made many curious and valuable remarks. He had taken a comprehensive view of the origin and progress of medicine, and of the various medical theories and opinions which have prevailed in different ages and countries; and seemed on this account peculiarly well qualified (independently of his practical knowledge) to have written a philosophical history of the science.

But the question was very different, how far he was well qualified to practise medicine with advantage as a lucrative profession; and the period was now arrived, when this point was to be determined. Several of his friends, although very doubtful as to the ultimate success of the measure which they recommended, were yet extremely desirous that he should try the effects of a regular profession; which they considered as affording the best prospect of giving an useful direction to his talents, and fixing his desultory habits. In deference chiefly to their opinion, he took his degree of doctor of physic at Cambridge in the year 1796, and had serious thoughts of commencing practice. But, after some hesitation, he wisely relinquished a design, which, whether successful or not, was unlikely to contribute to his happiness. His desires were moderate, and his private fortune exempted him from the necessity of following any employment as the means of subsistence.

subsistence. He was at liberty, therefore, to indulge his own inclinations; and his careless, independent habits of life, no less than the general cast of his character and understanding, rendered him altogether averse to the drudgery and restraints of a profession. It may be observed also as a circumstance by which he was undoubtedly much influenced in adopting this resolution, that he had suffered very greatly, during his attendance at the hospitals, in consequence of the acute and painful emotions he had constantly experienced from those sights of hopeless misery which he had so often occasion to witness. He justly apprehended that the frequent recurrence of such scenes, unavoidable in medical practice, would be destructive of his comfort and happiness.

The keen and exquisite sensibility, from which these feelings originated, was a striking feature in Mr. Tennant's character, and not only gave a colour to many of his opinions, but powerfully influenced his conduct. An instance of his practical benevolence, derived from this principle, happened about this period, which deserves to be mentioned. He had a steward in the country in whom he had long placed implicit confidence, and who was considerably indebted to him. In consequence of this man's becoming embarrassed in his circumstances, Mr. T. went into the country, to examine his accounts. A time and place were appointed for him to produce his books, and shew the extent of the deficiency; but the unfortunate steward felt himself unequal to the task of such an explanation; and in a fit of despair put an end to his own existence. Touched by this melancholy event, Mr. T. used his utmost exertions for the relief and protection of the family, not only forgave them the debt, but afforded them pecuniary assistance, and continued ever afterwards to be their friend and benefactor.

During the course of the year 1796, Mr. Tennant communicated to the Royal Society his paper on the Nature of the Diamond. Sir Isaac Newton had conjectured that this body was inflammable, as was afterwards proved by the experiments of the Duke of Tuscany and of Messrs. Darcet and Rouelle. M. Lavoisier effected its combustion by means of a lens, in close vessels, and obtained from it a gas, which precipitated chalk from lime-water. But this was at an early period of pneumatic chemistry; and although he concluded that the gas was fixed air, yet he did not consider the analogy between charcoal and the diamond as very intimate, but as depending only on their common property of being combustible. The merit of completely ascertaining the nature of this substance was therefore reserved for Mr. Tennant. He succeeded in burning the diamond when reduced to powder, by heating it with nitre in a gold tube. A solution of the alkaline salt was then poured into liquid muriate of lime; and the quantity of carbonic acid which had been generated was inferred from the weight of the precipitate, which was found to consist of carbonate of lime.

From experiments made upon minute quantities of diamond powder, not exceeding $2\frac{1}{2}$ grains, he shewed, by comparing them with Lavoisier's experiments on charcoal, that equal weights of diamond and charcoal yield equal quantities of fixed air, and that fixed air contains between 27 and 27·8 per cent. of diamond; results which very nearly agreed with those of M. Lavoisier, and were subsequently confirmed by the investigation of Messrs. Allen and Pepys.

In the course of his investigation of the diamond, Mr. Tennant observed that gold and platina were corroded and dissolved by heated nitre; and that on the addition of water to the salt, the metals, owing to the presence of nitrite of potash, were in a great measure precipitated. These appearances, together with some peculiar properties of the nitrous solution of gold, were the subject of a further communication to the Royal Society in 1797.

It is worthy of remark, that Mr. Tennant had ascertained the true nature of the diamond some years before he made the above communication to the Royal Society. In conversing about this time with a particular friend, whom he was attending with affectionate care during a lingering illness in the spring of 1796, he happened to mention this discovery. His friend, who had often lamented Mr. T.'s habits of procrastination, urged him to lose no time in making his experiment public; and it was in consequence of these entreaties that the paper on the diamond was produced. A still more remarkable example of the same indolence or inattention occurred in the case before alluded to of the paper on double distillation, communicated to the Royal Society in 1814, the substance of which he had mentioned to some of his friends during his residence at Cambridge.

These facts are memorable and instructive instances of the strength and weakness of Mr. Tennant's mind. His curiosity and activity were incessant. His memory was a great storehouse of discoveries and ingenious trains of reasoning. These he was continually treasuring up, with the intention of reducing them to order and preparing them for use at a more convenient season. But that period rarely arrived. In the carelessness of intellectual wealth, he neglected the stores of knowledge which he had accumulated, and suffered them to remain useless and unproductive, till his attention was recalled to them, perhaps after a long course of years, by some new fact or discovery, some remark in conversation, or other accidental occurrence. It is yet to be ascertained, by a careful examination of his papers, whether any fragments of this great body of knowledge still remain, which can now be converted to use. But there is too much reason to believe that the far greater part of them existed only in the mind of their author, and that with him they have unfortunately perished!

The state of the continent prevented Mr. Smithson from completing his education by the customary grand tour, and his distressing situation, from sea-sickness, was an effectual bar against a transatlantic voyage. It may, therefore, be readily conceived, that, with an independant fortune, a fondness for science, with an active mind, he must sometimes have been in want of a pursuit. This, and perhaps an opinion that his income might be further improved, proved, induced him to listen to a proposal of purchasing some allotments of an inclosure in Lincolnshire.

From the time of making his first purchase, Mr. Tennant paid great attention to the study of rural economy; and his attachment to this new pursuit gradually increasing, he became desirous of engaging in some agricultural concern upon a more extensive scale. With this intention, about the year 1798 or 1799, he purchased a considerable tract of waste land, newly allotted under an Enclosure Act, on the Mendip Hills in Somersetshire. The purchase was originally made in conjunction with a particular friend, who, for some time, resided on the spot, and personally superintended the concern. But a partition of the estate afterwards taking place, a portion of land was assigned to Mr. Tennant, situated near the well-known village of Cheddar, which was retained by him in his own hands, and became the principal scene of his farming operations. Here he built a small house, at which, during the remainder of his life, he passed some months every summer, besides occasional visits at other times of the year.

London, however, still continued to be the principal place of his residence. It must be obvious, that this must have obliged him to leave his farming concerns to agents. For a certain period (as it was reasonable to expect), owing partly to his own inexperience, and partly to unfavourable seasons, and other accidents, his speculations were not prosperous; and he occasionally suffered some anxiety and disappointment. But in process of time he acquired more practice and information, and became insensibly habituated to many trifling vexations, which at first had given him uneasiness. The prospect gradually brightened; and during the latter part of his life his concerns were brought into better order, and appear to have been attended with a reasonable degree of success.

The change in his habits, occasioned by his agricultural engagements, was not equally favourable to his scientific pursuits. His spirits were often exhausted, and his mind fatigued and oppressed, by the attention which he thought it necessary to bestow upon the correspondence with his agents, the examination of his farming accounts; and other details equally tedious and minute; and it is impossible to reflect upon the time thus consumed, without lamenting that it was not employed for purposes more beneficial to mankind, and more worthy of his genius and understanding.

It appears, however, from various notes and memoranda which are found among his papers, that he was very industrious in procuring information from the best works upon farming, and that he made various practical remarks during his journies, and collected many accurate and circumstantial details relative to the modes of cultivation adopted in different parts of England. In the course of these inquiries, he had discovered that there were two kinds of limestone known in the midland counties of England, one of which differed from common lime-stone in yielding a lime injurious to vegetation. He explained the cause of this difference in a paper communicated to the Royal Society in the year 1799; shewing that carbonate of magnesia is an ingredient in the latter

 species species of lime-stone, which he describes as an extensive stratum in the midland counties, and as being found also in many other situations, particularly among the primitive marbles, under the name of Dolomite. He gives the proportions in which lime and magnesia exist in many specimens of this lime-stone, and infers, from its slow solution in acids, and from its crystalline structure, that it is rather the result of chemical combination than of a casual mixture of the two earths. This conjecture has since been verified by the goniometrical researches of Dr. Wollaston, and by the near agreement of Mr. Tennant's analysis with the constitution of the mineral, as inferred from the law of definite proportions.

Mr. Tennant had found that grain will scarcely germinate, and soon perishes, in moistened and perfectly mild carbonate of magnesia; and that the injurious effects of the magnesia in agriculture do not depend on its property of long remaining caustic, but, probably on some inherent quality of the earth itself. He also made many experiments on the germination and growth of various seeds and plants in different mixtures of the simple earths; and, transporting portions of soil, either from his own estates, or from different parts of England, to the neighbourhood of London, he tried, on a small scale, the effects of various manures in promoting the growth of different vegetables, and endeavoured in this way to obtain hints for improved modes of cultivation.

In the year 1802, he communicated to the Royal Society his chemical examination of Emery, which had hitherto been considered as an ore of iron. He showed that it consists principally of alumina, and that it nearly agrees with the Corundum of China, which had been analyzed a short time before by Klaproth.

In the month of July during the same year, in endeavouring to make an alloy of lead with the powder which remains after treating crude platina with aqua regia, he observed remarkable properties in the powder, and found that it contained a new metal. But, while he was engaged in pursuing this investigation, the attention of two French chemists was accidentally directed to the same object. In the autumn of 1803, M. Descotils had discovered that the powder contains a metal which gives a red colour to the ammoniacal precipitate of platina; and M. Vauquelin, having treated the powder with alkali, obtained from it a volatile oxide, which he considered as belonging to the same metal.

In the spring of the year 1804, Mr. Tennant, having completed the course of his experiments, communicated the results to the Royal Society. He shewed that the powder consisted of two new metals, to which he gave the names of Iridium and Osmium, and that these might be separated from one another by the alternate action of heated alkali and of acid menstrua. By crystallization from the acid solution, he obtained a pure salt of iridium, from which he determined with accuracy the real properties of the metal and of its compounds; and, from a comparison with these, he ascertained that the volatile oxide belonged to another metal (osmium), which he also obtained in a state of purity.

The analysis of crude platina presented, perhaps, some of the greatest difficulties with which chemistry had ever yet ventured to contend. Besides affording traces of several of the known metals, the ore contained, in very minute quantities, four new metallic elementary bodies, whose existence was previously unsuspected, and whose respective characters were to be distinguished before the separate nature of the bodies could be ascertained. Dr. Wollaston and Mr. Tennant, who were employed upon this ore at the same time, and whose habits of friendly intercourse led them to communicate freely with each other during the progress of their experiments, gave proofs of industry, ingenuity, and accuracy, which has been acknowledged by every philosopher in Europe: Mr. Tennant in the manner already described, and his friend by the discovery of the two metals called Palladium and Rhodium.

On the 30th of November, 1804, Mr. Tennant had the honour of receiving the Copleyan medal, conferred on him by the Royal Society, for his various chemical discoveries.

About the year 1805 and 1806, Mr. Tennant made two journies, during successive summers, into Ireland, going and returning on both occasions by Scotland, in order to abridge, as much as possible, the sufferings attendant on a sea voyage. In the course of these journies, he visited most parts of Ireland which possess any attraction for a traveller, and had the advantage of viewing the Giant's Causeway, in company with Dr. Wollaston, whom he met by a fortunate accident in the north of the island.

His attention was extended to practical objects. He made many remarks on the agriculture, manufactures, and general state of Ireland. He was particularly struck with the vast population of the country, compared with that of Scotland, through which he had lately passed, and even with the average population of England. The backward state of its improvement and cultivation, considering its various resources, and the natural fertility of the soil, presented other objects of contrast, which could not fail to interest him. His attention was naturally led to the causes of this inferiority; but to enter into his opinions on this important subject, would require a wider field of discussion than is consistent with the limits of the present narrative. One observation, however, selected from many others, which is strongly marked with his characteristic liberality and good sense, may, perhaps, deserve to be mentioned. His curiosity had led him to inquire into the state of education among the lower Irish Catholics, and he was much struck with the narrow and illiberal cast of most of their books of popular instruction. This, which he justly considered as a serious evil, he attributed in a great degree to a sort of party spirit in the Catholic clergy, which arises from the unfortunate alienation subsisting between them and the government. He observed, that, if proper means were taken to conciliate this body, they would gradually relax from their strictness, and become better informed and more enlightened; and that thus, without the formality of conversion, the great mass of their followers might, perhaps, by little little and little, imbibe a portion of the spirit of protestantism, and be brought to partake of the knowledge and improvements of the age.

In one of the journies to Ireland which has given occasion to these remarks, Mr. Tennant was accompanied by Mr. Browne, the celebrated African traveller, with whom he had lived for some time in habits of great intimacy. As Mr. Browne, although not much personally known, was remarkable on several accounts, and a man of considerable merit, it may not be improper to describe shortly the nature and origin of this connection; which will also afford an opportunity of throwing a new light upon Mr. Tennant's character, and placing it in a particular point of view, in which it has not yet been considered.

Mr. Tennant was one of the most determined advocates of civilized life, in opposition to those sentimental theories, which extol simplicity, and undervalue the advantages of a refined and cultivated age. The decided superiority of the latter was one of his favourite topics of conversation; and he would often dwell with particular pleasure upon the spirit of improvement displayed in our own times, and the energy and intelligent activity of modern Europe, which he was fond of contrasting with the apathy and torpor of the East. Yet, by one of those inconsistencies from which no human understanding is entirely exempt, he took a singular interest and delight in all accounts of Oriental nations, and in the peculiarities and details of their characters, habits, and institutions. The historical recollections and images of ancient renown, which are associated with those remote countries, the entire difference of manners and religions, and the dignified gravity and imposing exterior of the present inhabitants, amused and gratified his imagination. He was a considerable purchaser and collector of books and engravings relative to the East; and had perused with great eagerness and curiosity all the numerous publications respecting Turkey, Egypt, and Persia, which have appeared during the last twenty years. He was also familiarly acquainted with the principal Eastern travellers of former times; among whom Chardin, Norden, Russell, and Shaw, were those whom he particularly valued. His knowledge of the countries described by these writers was remarkably accurate and minute, and extended in many cases even to geographical details.

With the tastes and feelings resulting from this turn of mind, it may easily be conceived that Mr. Tennant had a peculiar gratification in the society of Mr. Browne. He found in that distinguished traveller, not only an intimate acquaintance with those countries which so much interested his curiosity, but a considerable fund of learning and information, united with great modesty and simplicity, and with much kindness of disposition. By strangers, however, Mr. Browne's character was apt to be misunderstood. Whether from natural temperament, or from habits acquired in the East, he was unusually grave and silent, and his manners in general society were extremely cold and repulsive. Even in company with

Mr.

Mr. Tennant, to whom he became sincerely attached, he would often remain for some time gloomy and thoughtful. But, after indulging himself with his pipe, his eye brightened, his countenance became animated, and he described in a lively and picturesque manner the interesting scenes in which he had been engaged, and to which he again looked forward. Of the impression left on Mr. Tennant's mind by these interviews, some idea may be formed from the following passage of a letter written by him to an intimate friend, soon after he had received the account of Mr. Browne's death. "I recall," he says, "the *Noctes Arabicæ* which I have often passed with him at the Adelphi, where I used to go whenever I found myself gloomy or solitary; and so agreeable to me were these soothing romantic evening conversations, that, after ringing his bell, I used to wait with some anxiety, fearful that he might not be at home."

In the autumn of 1812, some years after his journey to Ireland with Mr. Tennant, Mr. Browne took his departure from England on an expedition, which he had long projected, to the unexplored Tartar city of Samarcand. He first visited Constantinople; and, at the instigation of Mr. Tennant, made a diligent but fruitless search for the meteoric stone which is mentioned to have fallen at Egospotamus, in the Parian Chronicle, and in Pliny. From Constantinople he went to Smyrna, where he passed the winter; and from thence to Tabriz in Persia; from both which places he wrote several very interesting letters to Mr. Tennant. In one of these he mentions, that in passing through Armenia he had satisfied himself, by mineralogical observations which he had made, that a considerable tract of that country, including Mount Ararat, is of volcanic origin. He likewise ascertained, by measuring the temperature of boiling water (at the suggestion of Mr. Tennant), that the city of Arzroum, the capital of Armenia, is 7000 feet above the level of the sea.

Most unfortunately for the cause of scientific discovery, Mr. Browne perished afterwards (as is well known) by the hands of banditti, near the river Kizzil Ozan, east of Tabriz. Previously to his leaving England, when he was setting out on this journey, he had made his will, by which he named Mr. Tennant one of the executors, and left him a considerable bequest. On opening the packet in which the will was enclosed, a paper was found, in Mr. Browne's hand-writing, containing a characteristic and remarkable passage from one of Pindar's Odes, highly expressive of that generous ambition, and contempt of danger and death, which are the true inspiring principles of such enterprizes.* Till he saw this paper, Mr. Tennant, notwithstanding his long intimacy, had never been fully aware of the real force of his friend's character, and of the powerful and deep feelings which his cold manners and habitual reserve had effectually concealed from observation.

* Pindari Olymp. Carm. 1, v. 129.

It

It was before stated, that Mr. Tennant's health had been gradually declining. His frame was naturally feeble; and during the latter years of his life, though he was seldom materially indisposed, he was scarcely ever entirely well. Almost always on going to bed, he had a certain degree of fever, and was often obliged to get up in the middle of the night, and to obtain relief by exposure to the cool air. To preserve himself in any degree of bodily vigour, he was under the necessity of using daily exercise on horseback; a practice which, though he complained of as a serious encroachment on his time, he hardly ever omitted in the severest weather. His long journies into various parts of England and Ireland were usually performed in this manner. Even about town he was frequently in the habit of transacting little concerns of business by that conveyance.

In the summer of 1809, as he was riding to Brighthelmstone, he met with a serious accident, by which his collar bone was broken. He was removed to the house of his friend Mr. Howard, in the neighbourhood of London, where he was treated with the most affectionate kindness, and remained till his complete recovery.

During the early period of his residence in London, Mr. Tennant had led rather a retired life; but in his more advanced years he went much more into the world, and cultivated general society. He had a particular pleasure in conversing with intelligent travellers newly returned from distant countries, or in suggesting rational objects of inquiry to such as were about to visit them. As an instance of the latter, it may be mentioned, that he was at considerable pains in instructing M. Burchardt, a gentleman sent out by the African Association to explore the interior of that continent, in the principles of mineralogy. He frequently gave small morning parties at his chambers in the Temple, which he rendered very amusing to his friends by the exhibition of interesting prints and drawings, of rare specimens and new substances in chemistry, or of other objects calculated to gratify an intelligent and well-directed curiosity. It happened, in the spring of 1812, that he had engaged to shew his mineralogical collection to a large party of his acquaintance, with a view of explaining to them the nature of some of the principal substances, and of giving them some general ideas of mineralogy. His intention being known, several others of his friends requested permission to be present; and he was gradually induced to extend his original plan, and to give a few lectures on the general outlines of mineralogical chemistry. The undertaking was somewhat arduous, considering the expectations which his high character was likely to excite, his total inexperience as a lecturer, and the difficulty of adapting himself to so mixed an audience, which, though consisting principally of females, included many individuals distinguished for rank, science, and literature. It was attended, however, with complete success. Mr. Tennant was seated on a music-chair, which enabled him to turn with facility to every part of an audience, so numerous, that many were obliged to stand. But, though his first lecture was protracted for nearly four four hours, no one shewed the least symptoms of weariness, nor would, probably, have required refreshments, had not his own gallantry reminded them of it, by sending to a neighbouring confectioners for ices and cakes.

The great clearness and facility of his statements, the variety and happiness of his illustrations, and the comprehensive philosophical views which he displayed, were alike gratifying to every part of his audience. He delivered about four lectures, each of which was of great length; yet the interest of his hearers were never in the least suspended. Though his style and manner of speaking were raised only in a slight degree above the tone of familiar conversation, their attention was perpetually kept alive by the spirit and variety with which every topic was discussed, by anecdotes and quotations happily introduced, by the ornaments of a powerful but chastised imagination, and, above all, by a peculiar vein of pleasantry, at once original and delicate, with which he could animate and embellish the most unpromising subjects.

The delivery of these lectures may be considered by some persons as a very trifling occurrence in the life of a man of science; but the writer has thought it well worthy of a place in the present narrative, not only as affording a new proof of the extent and variety of Mr. Tennant's powers, but because it exemplifies in a very pleasing manner one of the striking and amiable peculiarities of his character. As he had a remarkably clear perception of the most refined scientific truths, so he possessed a power of explaining and illustrating such subjects, which might justly be deemed unrivalled. Without any ostentation of learning, he had a peculiar delight in communicating knowledge to others; but especially in unfolding the principles of science to young persons, capable and desirous of receiving such information; and he partook with the most lively sensibility in the emotions of curiosity, pleasure, and surprise, which his lessons seldom failed to inspire. He entirely differed from those who condemn, as trifling and superficial, that increasing taste for scientific knowledge in the higher and more opulent classes, to which many circumstances have lately contributed. He considered the diffusion of this taste among the young, the idle, and the wealthy, though liable, in some cases, to degenerate into affectation, as being in its general effects highly beneficial; both by affording to an important class of society additional sources of intellectual pleasure and new objects of rational pursuit; and indirectly, by obtaining for science and its professors a greater degree of public consideration and respect than they have enjoyed in any former age.

In the spring of 1813, Mr. Tennant delivered to the Geological Society a lecture on the principles of mineralogy, in which he illustrated several artificial productions analogous to those of nature. In the same year, the chemical chair in Cambridge became vacant. The eyes of all were instantly turned on Mr. Tennant. At one time, another name was hinted, but no longer heard when Mr. T.'s intentions were known. It is impossible to say what would be the advantages derived from so much genius, such powers of elocution, such a fondness for his subject, and so popular a mode of expressing himself in an university crowded with men of leisure, and in an age devoted to experimental philosophy. We can only add, that during the only course he delivered, the theatre was at every succeeding lecture more crowded, and the recollection of what he taught is still recent with his hearers, and will probably be so as long as they live.

In June, 1814, his two last communications were read before the Royal Society, which we have lately given in our Journal.

We come now to an era in Mr. Tennant's life on which we are obliged to dwell with some minuteness, though the recollection of every part is painful, as all seems connected with one distressful event. We have already remarked the impediments to his early wishes of exploring foreign parts. He had always lamented his omission to visit the continent of Europe during the short peace of 1802. He therefore took an early opportunity, after the general pacification of 1814, of passing over to France, for the purpose of observing those changes which the eventful period of the last twenty years has produced, and of renewing his personal communications with men of science at Paris, from which he had been so long debarred. His own experience had taught him how much may be known, which has not been communicated in books. In this respect he was not disappointed; for in one of his letters, written a short time before he left Paris, he mentioned with much satisfaction how many interesting facts he had collected which would enrich his Cambridge lectures. The following passage of a letter, in which he relates his first sensations, may be worth transcribing, both because it affords somewhat of a specimen of his general manner, and may, perhaps, recall to the recollection of his friends several of his favourite topics and opinions. "After a short and favourable passage of three hours and a half, we got into the harbour of Calais, with its immense pier. The difference of every thing struck me prodigiously. I felt quite intoxicated with the novelty of the scenery, the abundance of the country, the bright blue sky without a cloud, and the broad magnificent roads, with elms, and sometimes fruit-trees, on each side, for fifty miles. I was a little mortified on comparing the climate with our own, till I observed the many points in which its advantages were neglected: open fields; harvest not finished, as in England; corn full of weeds; and oats more than one-third inferior in quality to my own at Mendip. On approaching Paris, the vineyards were new features of this superior climate. At Paris, what strikes one most is the narrowness of the streets, along which, as I passed, I was in constant expectation of getting out of the eternal narrow lanes. Now I am quite reconciled to them.—The backward state of civilized life is more apparent here than in the country. You are struck with the imperfection of every thing, and the mixture of dirt and meanness with pomp and expence. In the theatres, (which, however, are quite inferior to ours in size, and still more in in elegance,) you see, in the passages behind the boxes, dirty pavements of brick, with wide cracks; and the boxes are opened by a few old women, who are employed during the intervals in knitting or mending stockings. The women are such as might be taken from a field in England, where they would be employed in weeding."

During his stay in France, Mr. Tennant, in the months of October and November, made a tour into the southern provinces, which he had not before seen, and visited Lyons, Nismes, Avignon, Marseilles, and Montpellier. He was much gratified by this journey, during which he made many interesting remarks on the state of the country, paying particular attention to mineralogy and agriculture. In his letters written about this time, he describes, in striking terms, the feelings of enjoyment which he always experienced from new scenes and objects, and especially from viewing the productions of Nature in southern climates. In speaking of the range of mountains from whence the Saone takes its rise, he says, "The country is the most rich and picturesque that can be imagined; but the contrast of the beggary and dirt of the towns and common habitations with the rich vegetation of the country is universal, with the exception only of towns of the first rate. I am not yet sufficiently at home in the political economy of the country to say on what this depends: in part, on its extreme population.—After passing these mountains, a new world appears, marked with the characters of a southern climate. The race of people is different, with finer skins than in the North. The country women wear immense hats, to defend them from the sun; and the houses (there being no snow) have low pitched roofs, like those in Poussin and Claude Lorraine. Nothing can be more beautiful than this style of building, which continues to the Mediterranean.—Proceeding south, vines and mulberries chiefly cover the ground; and following the Saone, its mountainous banks, studded with country houses, almost buried in the rich vegetation of figs, mulberries, vines, and pomegranates, exceed all anticipation. Suppose the scenery of the hot-wells at Bristol extended 50 miles, with a broader rapid river, higher mountains, under a glowing climate, and thickly set with white cottages, intended to be copied by a painter. Nothing is so fine as the situation of Lyons, and the views into the paradisiacal valley from the mountains on each side of the town. This wonderful scenery continues along the Rhone by Vienne (from whence comes the Coterotie wine) and Tain (from whence Hermitage) till you arrive in Provence, where the olive, a new production of the southern climates, begins to make its appearance. Through this rich garden or forest, you come to the calcareous mountains, which on their summits are white rocky hills, covered with wild plants, thyme, rosemary, lavender, ilex, quercus coccifera, and innumerable plants unknown in our latitude, but which I hope to raise in England next year, to renew my impressions of this country. These mountains enclose the valley of that wonderfully situated immense town of Marseilles. As you

 descend, descend, the Mediterranean appears, and the great city, with its endless suburbs, in the hollow vallies sloping towards the sea. * * * * It was with infinite regret that I left Marseilles: if I could stay the winter, it should be there. Avignon is pleasingly situated; Nismes has fine antiquities; Montpellier is in a rich and plentiful country; but they are all *triste* and dead compared with Marseilles, where every attraction is united."

On his return from Montpellier to Paris, he writes, while stopping for a few days at Lyons,—"At Montpellier I had the peculiar advantage of a most attentive acquaintance (M. Berard), who is one of the best chemists in France. The country affords few such; but he was brought up at the feet of Berthollet, who gave me a letter to him. He succeeded to the chemical works of Chaptal, which are now very extensive, and carried on with great intelligence.—On my return, I visited the great Roman aqueduct of the Pont du Gard, so striking for its antiquity, its altitude, its solidity, and the very romantic situation where it passes over the valley and river. Pont St. Esprit is hardly less interesting, being of such immense extent (more than half a mile), and the lowest bridge on the Rhone. It was built, not by the Romans, but in the darkest ages, by the powers of superstition, the great principle of energy and exertion at that period. In 1265, the offerings to the convent of St. Esprit were sufficient for this undertaking, and were thus applied by the monks, with honour to themselves, and with great advantage to a remote posterity; for the passage over it is immense at this time.—I stopped for half a day at Tain, from whence we have the Hermitage wine. Nothing can be more beautiful than the gold colour of the 'vine-covered hills,' nor more extraordinary than the sand or gravel in which the plant grows. There is not a particle of soil, but merely broken sharp fragments of granite, chiefly felspar, perfectly clean; for, though the roots of the vine are manured once a year, this totally disappears. The gravel soil is supported by walls. From the top are seen endless mountains, which border on the Rhone, and which, along the slopes facing the south, are yellow with vines, in spite of the extreme barrenness of the soil. Such mountains, which are here among the most valuable parts of the kingdom, would not in England be worth a penny an acre.—From this place (Lyons) we rode the other day into the mountains near Arbresle, to see a new copper-mine, consisting of a blue carbonate, compared with which, the specimens from Siberia are quite insignificant. * * * *."

Mr. Tennant returned to Paris during the month of November, and meant to have seen England about the latter end of the year. But he continued to linger on till February following. On the 15th of that month, he reached Calais. On the following day, he wrote to explain the cause of his long delays, occasioned, he said, "by his postponing the disagreeable exertion of setting off, added to the severe weather, and the odious view of the ocean, of which he had so great a horror, that it darkened the agreeable prospect of meeting his friends in England."

The

The wind then blew directly into Calais harbour, and continued unfavourable for several days. After waiting till Monday the 20th, he went to Boulogne, in order to take the chance of a better passage from that port, in company with Baron Bulow, a German officer, who was also going to England. They embarked on the morning of Feb. 22d, but the wind was still adverse, and blew so violently that the vessel was obliged to put back. When Mr. Tennant came on shore, he said, "that it was in vain to struggle against the elements, and that he was not yet tired of life." Alas! how little we know of futurity. How little did he expect that his very caution would prove the means of a death, if possible, more sudden, certainly less expected, than from the ocean itself!

It was determined that, in case the wind should abate, another trial was to be made in the evening. During the interval, Mr. Tennant proposed to the Baron that they should hire horses, and take a ride. They rode at first along the sea side; but at Mr. Tennant's suggestion, they went afterwards to Bonaparte's Pillar, which stands on an eminence about a league from Boulogne. Having been to see it himself the day before, he was now desirous of shewing it to Baron Bulow.

On their return from thence, they deviated a little from the road, in order to look at a small fort near the pillar, the entrance to which was over a fosse 20 feet deep. On the side towards them there was a standing bridge for some way, till it joined a draw-bridge which turned upon a pivot. The end next to the fort rested on the ground. On the side towards them it was usually fastened by a bolt; but the bolt had been stolen about a fortnight before, and by an unpardonable neglect was not replaced.

As the bridge was too narrow for them to go abreast, the Baron said he would go first, and attempted to ride over it. But, perceiving that it was beginning to sink, he made an effort to pass the centre, and called out to warn his companion of the danger; but it was too late—they were both precipitated into the trench. The Baron, though much stunned, fortunately escaped without any serious hurt; but, on recovering his senses, and looking round for Mr. Tennant, he found him lying under his horse, nearly lifeless.

He was first conveyed to a cottage, inhabited by the person who had the care of the pillar. Medical assistance was instantly procured from Boulogne, when it was found that his skull was dreadfully fractured, as to leave no hope of recovery. One of his arms also was broken. He was taken to the Civil Hospital, as the nearest and most convenient place to receive him, and, after a short interval, seemed, in some slight degree, to recover his senses, and made an effort to speak, but without effect, and died within an hour.—His remains were interred a few days afterwards in the public cemetery at Boulogne, being attended to the grave by most of the English residents.

Thus perished, at the full maturity of his judgment, in the possession of all he wished for in this life, and in the full capacity of enjoying all, one of the clearest genius, the faithfullest experimentors, mentors, and the readiest communicators, that has appeared in an age which can boast a Woolaston, a Davy, besides a host of other illustrious foreign and domestic names!

Mr. Tennant was in his 56th year when he died. His person was tall and slender. He was long visaged, with light hair and complexion. His countenance in early life had been singularly engaging; and, at favourable times, when he was in good spirits and tolerable health, was still very pleasing. The general cast of his features was expressive, and bore strong marks of intelligence. Several persons have been struck with a general resemblance in his countenance to the well-known portraits of Locke.

It has been remarked by some, that Mr. Tennant was more frugal than might have been expected from his general character. If this was really the case, it only shows what is every day proved that nothing human is altogether accordant with our ideas of perfect consistency. That he was not niggardly, the anecdote already given is a sufficient proof. But there was certainly an inattention to those wants which perplex common minds more than they ought. On one occasion, a few friends breakfasted with him at his chambers in the Temple. The number of tea-cups were insufficient. When Mr. T. complained of this, his servant answered, with much composure, that all the others were filled with his stuffs (experiments). Mr. T. replied, with the same calmness, well, buy some more, at the same time taking a few half-pence from his pocket.

The leading parts of his moral and intellectual character are apparent in the principal transactions of his life. But in this memorial, however imperfect, of the talents and virtues of so extraordinary a man, some attempt must be made to delineate those characteristic peculiarities, of which there are no distinct traces in the preceding narrative.

Of his intellectual character, the distinguishing and fundamental principle was good sense; a prompt and intuitive perception of truth, both upon those questions in which certainty is attainable, and those which must be determined by the nicer results of moral evidence. In quick penetration, united with soundness and accuracy of judgment, he was, perhaps, without an equal. He saw immediately, and with great distinctness, where the strength of an argument lay, and upon what points the decision was ultimately to depend; and was remarkable for the faculty of stating the merits of an obscure and complicated question with brevity, simplicity, and precision. The calmness of his temper, as well as the singular perspicuity, which he displayed on such occasions, seldom failed to convince the unprejudiced, and to disconcert or silence his opponents.

These powers of understanding were so generally acknowledged, that great deference was paid to his authority, not only upon questions of science, but upon most others of general interest and importance. What Mr. Tennant thought or said upon such subjects, his friends were always anxious to ascertain; and his opinions had that species of influence over a numerous class of society which is the most certain proofs of superior talents.

Next

Next to rectitude of understanding, the quality by which he was most distinguished, was a lofty and powerful imagination. From hence resulted a great expansion of mind, and sublimity of conception, which, being united with deep moral feelings, and an ardent zeal for the happiness and improvement of mankind, gave a very peculiar and original character to his conversation in his intercourse with familiar friends. He partook with others in the pleasure derived from the striking scenes of nature; but was more particularly affected by the sight or contemplation of the triumphs of human genius, of the energies of intelligent and successful industry, of the diffusion of knowledge and civilization, and of whatever was new and beautiful in art or science. The cheerful activity of a populous town, the improvements in the steam-engine, the great Galvanic experiments, and, above all, the novelty and extent of the prospects afforded by that revolution in chemical science which has illustrated our own age and country, excited in him the liveliest emotions, and called forth the most animated expressions of admiration and delight.

This keen sensibility to intellectual pleasure may be partly understood, from the following passage of a letter written by him in January 1809, to an intimate friend who was then abroad. After mentioning the great phenomena of the decomposition of the alkalies by Voltaic electricity, and giving a general view of the experiments founded upon them, he thus concludes: "I need not say how prodigious these discoveries are. *It is something to have lived to know them.*"

His taste in literature and the fine arts partook, in a considerable degree, of the peculiar character of his imagination. His favourite writers (those whom he most valued for the eloquence of their style) were such as describe "high actions and high passions," and have the power of exciting strong and deep emotions. Of the poets, he principally esteemed Virgil, Milton, and Gray; and the prose writers, to whom he gave the preference for powers of composition, were Pascal and Rousseau. He had a particular admiration of the "Pensées de Pascal," regarding it as a production altogether unequalled in energy of thought and language, in occasional passages of refined and deep philosophy, and, above all, in that sublime melancholy, which he considered as one of the peculiar characteristics of great genius.

The same principles governed Mr. Tennant's judgment in the fine arts. Considering it as their proper office to elevate the mind, and to excite the higher and nobler passions, he estimated the merits of the great masters in music and painting by their power of inspiring these emotions. What he particularly admired in musical compositions, was that tone of energy, simplicity, and deep feeling, of which the works of Handel and Pergolesi afford the finest specimens. In painting, he awarded the superiority to those distinguished masters, of whom Raphael is the chief, who excel in the poetical expression of character, and in the power of representing, with spirit, grace, and dignity, the most exalted sentiments and affections.

It was almost a necessary consequence of his intense and deep feeling of these higher beauties, that his taste was somewhat severe, and that his ideas of excellence, both in literature and the fine arts, were confined within strict limits. He totally disregarded mediocrity, and gave no praise to those inferior degrees of merit, from which he received no gratification.

In consequence, principally, of the declining state of his health, his talents for conversation were, perhaps, less uniformly conspicuous during his latter years. His spirits were less elastic, and he was more subject to absence or indifference in general society. But his mind had lost none of its vigour, and he never failed, when he exerted himself, to display his peculiar powers. His remarks were original; and his knowledge, assisted by a most retentive memory, afforded a perpetual supply of ingenious and well-applied illustrations. But the quality for which his conversation was most remarkable, and from which it derived one of its peculiar charms, was a singular cast of humour, which, as it was of a gentle equable kind, and had nothing very pointed or prominent, is hardly capable of being exemplified or described. It seldom appeared in the direct shape of what may be called *pure* humour, but was so much blended either with wit, fancy, or his own peculiar character, as to be, in many respects, entirely original. It did not consist in epigrammatic points, or brilliant and lively sallies; but was rather displayed in fanciful trains of imagery, in natural, but ingenious and unexpected, turns of thought and expression, and in amusing anecdotes, slightly tinged with the ludicrous. The effect of these was much heightened by a perfect gravity of countenance, a quiet familiar manner, and a characteristic beauty and simplicity of language. This unassuming tone of easy pleasantry gave a very peculiar and characteristic colouring to the whole of his conversation. It mingled itself with his casual remarks, and even with his graver discussions. It had little reference to the ordinary topics of the day, and was wholly untinctured by personality or sarcasm.

It should be mentioned, among the peculiarities of Mr. Tennant's literary taste, that, in common, perhaps, with most other original thinkers, he bestowed little attention on books of opinion or theory; but chiefly confined himself to such as abound in facts, and afford the materials for speculation. His reading, for many years, had been principally directed to accounts of voyages and travels, especially those relating to Oriental nations; and there was no book of this description, possessing even tolerable merit, with which he was not familiarly conversant. His acquaintance with such works had supplied him with a great fund of original and curious information, which he employed with much judgment and ingenuity, in exemplifying many of his particular opinions, and illustrating the most important doctrines in the philosophy of commerce and government.

Of his leading practical opinions, sufficient intimations have been given in the course of the preceding narrative. They were of a liberal and enlightened cast, and such as might be expected from the the character of his genius and understanding. Among them must be particularly mentioned an ardent but rational zeal for civil liberty, which was not, in him, a mere effusion of generous feeling, but the result of deep reflection and enlarged philosophic views. His attachment to the general principles of freedom originated from his strong conviction of their influence in promoting the wealth and happiness of nations. A due regard to these principles he considered as the only solid foundation of the most important blessings of social life, and as the peculiar cause of that distinguished superiority, which our own country so happily enjoys among the nations of Europe.

Of his moral qualities, it is scarcely possible to speak too highly. He described himself as naturally passionate and irascible, and as roused to indignation by any act of oppression or wanton exercise of power. The latter feeling he always retained, and it formed a distinguishing feature of his character. Of his irritability, a few traces might be occasionally discovered; but they were only slight and momentary. His virtuous disposition appeared on every occasion, and in every form, which the tranquil and retired habits of his life would admit. He had a high sense of honour and duty; and was remarkable for benevolence and kindness, especially towards his inferiors and dependants. His merits were most conspicuous in the intercourse of social life. His amiable temper, and unaffected desire of giving pleasure, no less than his superior knowledge and talents, had rendered him highly acceptable to a numerous and distinguished circle of society, by whom he was justly valued, and is now most sincerely lamented. But the real extent of his private worth, the genuine simplicity and virtuous independence of his character, and the sincerity, warmth, and constancy of his friendship, can only be felt and estimated by those to whom he was long and intimately known, and to whom the recollection of his talents and virtues must always remain a pleasing, though melancholy, bond of union.

We have selected from several sources this interesting biography; but we are more particularly indebted to *Dr. Thompson's Annals of Philosophy.*

---

"The following very singular occurrence happened in the year 1759, at Chelwood, a village near Pensford (Somerset):—The sexton of the place opened a grave in which a man, who died of the small-pox, had been interred near thirty years before. The coffin was of oak, and so firm, that it might have been taken out whole; but the man forced his spade through the lid, when there issued a most nauseous stench. The person who was to be buried being of eminence, most of the inhabitants of the village attended the funeral: in a few days afterwards, fourteen persons were seized with the small-pox in one day; and in three days after, all but two in the whole village, who had not had it, were seized in like manner. It is remarkable, the disease was of so favourable a nature, that no more than two persons died of it."—*Walpole's British Traveller*, edit. 1784, p. 365.

